# Treatment of Proximate Surfaces

**Published:** 1889-04

**Authors:** Safford G. Perry


AMERICAN JOURNAL

—OF—

DENTAL SCIENCE.

VOL. XXII. THIRD SERIES—APRIL, 1889. NO. 12.

ARTICLE I.

### TREATMENT OF PROXIMATE SURFACES.

BY SAFFORD G. PERRY, D. D. S.

[Read before the Odontological Society of Pennsylvania, December 13, 1888.]

Some years ago I made a careful estimate from one of my chart books, and found that more than half my time had been given to the care of the proximate surfaces. It is probable that my experience has been so nearly that of others that it is safe to say that in practice to-day the care of these surfaces requires more thought and effort than all other dental work combined. With reference to the treatment of buccal or grinding surfaces there can be little difference of opinion. Methods may differ, but results must be the same.

But when we come to consider the treatment of proximate surfaces, the simple conditions are replaced by complex ones that are so various, that one must have had many years' experience to be able to master them in all their details.

The first and most important question that is presented to the mind of every man who undertakes the care of a proximate surface is the question of contour. There can be no evasions of this question by one who desires to be equipped for the best work of the present day.

I will not pause here to go fully over the ground of the old discussion of so-called permanent separations *versus* restorations. I may as well state plainly at the outset that I am an uncompromising advocate of the restoration of the shapes of the teeth, as a general rule of practice. The reasons for this are so many that a long session would be required to relate them in detail; and, moreover, they are so self-evident that it would seem to be a waste of time and an affront to your intelligence to go over them. And yet, as hinted by your committee, after all that has been so conclusively written against the pernicious habit of filling, and all that has been done in practical work by so many of our best operators in proof of the advantages of restorations, it still remains with many an open question, and to-day, throughout the world, if men's opinions are to be judged by their works, there are a very large number of operators, —many of whom we all know and respect, and some of whom we love,—who are still so evidently bound by habit to the unconscious dangers and temptations of the old practice of filing that it would almost seem as if we were yet in the babyhood of our profession, so that, after all, it seems as if I must pause a moment to touch upon the dangers of the one practice and the advantages of the other, even if I betray my "bias" to the point of weakening my argument to your minds. This "bias" is not of recent date. It is a slow growth, and as inevitable and unalterable as the conviction that two and two make four. In astronomy the "personal equation" has always to be taken into account. Through powerful glasses, that greatly magnify the slightest variation of vision, no two persons can be found who can see and describe the heavenly bodies exactly alike. And so in our field of effort it cannot be expected that all should see with the same eyes, and differences of opinion must be expected on most subjects; but on this, which it seems to me is capable of distant definition, I fail to see how there can be, if we really understand each other, a very wide difference of opinion.

Our opinions are the result of accumulated experiences, and one who has had long experience may perhaps lay claim to the privilege of speaking with some degree of confidence on such a subject as this.

If I venture to sketch my experience in justification of my convictions on this subject, I trust it will be considered a legitimate means of argumentation, and not an egotistic desire to thrust myself into the subject. And it will be the only justification for the dogmatic tone this paper may assume.

In order to make the points I desire to make, I must refer to and quote from a paper of mine on the subject, read in this city before the Odontographic Society of Pennsylvania, in 1870. In that paper I advocated the perfect restoration and, in some cases, the slight exaggeration of the shapes of the teeth. This will be shown by the following quotation:

"If we take nature as our guide, and desire to attain to her perfection, it is evident that, between molars and bicuspids at least, the file should only be used as it may facilitate the operation of restoring with gold the lost shape of the tooth. * * * If there must be a departure from the natural form of the teeth, I would prefer in such cases the other extreme—that of leaving the gold in proximate cavities slightly projecting, so that it, and not the tooth, shall rest against the adjoining tooth, or the filling in the adjoining tooth, as the case may be. * * ° I do not wish to be understood as advocating the building out of rounded knobs of gold between the teeth, so that they shall be held apart; but I would ask your attention to the propriety of packing the filling a little beyond the original outline of the tooth and finishing up with thin, trowel-shaped files and sickle-shaped scrapers, leaving the gold, when finished, *quite* as full as the tooth originally was, rather than pass between them a separating file, that would leave a flat, proximate surface. I should prefer a slightly unnatural fulness, rather than have a space, or an unnaturally flat surface."

The point I wish to make by this quotation, and which I cannot make in any other way, is, that a very large proportion of the proximate fillings made on this general plan before this was written are standing to-day, after twenty years' use, in good condition.

This is stated in no boastful spirit, but with a desire to present the exact facts. A very large proportion of the fillings made several years later, when I had become a convert to Dr. Arthur's system, have since been removed, many of them at great cost of time and labor, owing to the difficulty of restoring the lost shapes of the teeth. If Dr. Arthur's book had never been published and I had not been influenced by his earnest personality, but had held to my early plan of restoration, my patients would have been saved endless annoyance, and I should have had no dark period in my professional life to look back upon. The publication of his book and the circumstances of making his personal acquaintance, followed by a long and interesting correspondence with him, led me step by step to look with favor on his system, until I came to the point of making all the spaces described by him. I became so demoralized as to willing destroy the shapes of the teeth that I had before guarded with jealous care.

The result of that practice finally became a source of discouragement and mortification to me. Failures which formerly occurred only in reasonable numbers, under this system became surprisingly frequent. Irritation of the gums, and change of position of the teeth, which could not occur before, so frequently followed this practice that, after enduring it a few years, I was glad to return to the old one of the restoration of the shape of the teeth. I have continued this practice to the present day, and I expect to continue it to the end. As I look back over the past twentyfive years I see so much to commend in it, and so little in the other, that there is left for me no other choice. Having been twice on one side of the question and once on the other, I feel entitled to speak with no little confidence.

In 1870 thc restoration of the shapes of the teeth was not common practice. There was little to encourage one in that practice at that time. It was not unusual for the older operators, in examining a large contour filling, to probe along the cervical border, and with an ominous shake of the head predict that failure would follow there in a few years. But as a matter of fact failures have not followed in any considerable number of cases, even after all these years. These operators, taught by their experience to expect failures at all the sheltered points, expected it here. They failed to see that a new set of conditions had been established by which decay was almost certain not to occur. But they could not be made to believe this, and it could not be proven until after many years—except to those who had the faith that is born of enthusiastic and irresistible eonviction.

Fortunately, during that period when Dr. Arthur's earnest words— for he was as earnest as he was conscientious —had unsettled my convictions, there were still many patients whose teeth I never touched only to restore them, and to-day they are the patients most creditable to me. Many of them have large contour fillings that cost pain and effort; but, once being done so that the teeth were held in position and the gums protected, the years come and go and the patients forget that they have teeth at all. This is no fancy picture, but can be verified at any time. In my own teeth are large contour fillings, described in the article from which I have quoted, and put in by Dr. Varney over twenty years ago. They stand untouched from the day they were finished, and can be seen to-day by any one who cares to examine them. They have been a priceless benefit and comfort to me, and they stand an eloquent example of his matchless skill and an unanswered argument in favor of the restoration of the shapes of the teeth.

It is only after a long stretch of time that a just estimate can be made of the relative value of these opposing systems, It is easy to see that at first a clear gain seems to be made by making permanent separations. It takes years before the evil is noticed. It was partly this delusion that led me to adopt it; and it required several years before I saw that I was treading on dangerous ground. It was at first a great satisfaction to cut between molars or bicuspids where a cavity was suspected, and to find it and fill it easily and quickly, and flatter myself that the proximate surfaces were laid open so that they could be easily examined, and yet the gum but little, if any, disturbed. I could not then anticipate the slow change of position of the teeth; the disturbance of the festoons of the gums, owing to the loss of the bulge of the teeth that nature had designed to protect them; the recurrence of decay near the gum, causing the melting away of the shoulder that had helped to hold them in position; the slipping of food through, against and under the gum; and finally, the need of large fillings, that must now be made under most unfavorable circumstances. With the teeth tipped, the gums disturbed and sensitive, and the cavity extending on the root of the tooth, the task is much harder than if the cavity had, when of fair size, been filled by cutting down from the grinding surface and making a contour filling which should be so shaped that it would stand firmly against the neighboring tooth, and of such size that its borders would be free, except along the cervical edge, when the teeth had settled to their proper place. If the cavity had been neglected until it had become large, if filling on the same plan, it would be still safer; because then the cervical border of the filling would have to go under the gum, and if properly done, no further anxiety need be felt for it.

If the teeth chanced to be of good structure and the cavity small, how much better to spread them with a separator the thickness of a sheet of thin sand-paper, and cutting with small burs, or specially shaped, delicate excavators, from the grinding, lingual or buccal aspect, fill them with gold or copper amalgam, and allow them to go back to their natural positions, with their contour absolutely untouched.

Following this plan, it would not be probable that a patient would come in saying, as one did to me some years after the Arthur aberration: "This space between my teeth is giving me no end of trouble and annoyance, and I think there must be a cavity there. I have named it 'Perry's Chasm.'" Nor would there have been made to me the following remark: "Doctor, do you notice that on the left side, where many years ago separations were made between the back teeth, nearly all the cut surfaces have had to be filled; while on the other side, where no cutting was ever done, the teeth have never been filled and are still in good condition."

Only a few days ago a lady said to me: "Do you remember that many years ago you wanted to cut between some of my back teeth because you thought there were or would be cavities there, and mother begged you not to do it, and it was not done; and do you see that all of those teeth are still sound? Mother knew; did she not?"

I tell you, gentlemen, mothers do know; the public knows, and you may reason with all the plausibility at your command and you cannot overcome the widespread, instinctive dislike of that practice. You may flatter yourself that you have made a good argument, and as the patient does not get up and leave at once, you go on with your cutting and filling. But a year goes by and your patient does not re-appear; he has quietly slipped away to some one of these men who try to leave the teeth as nature made them.

The public calls this filing "taking off the enamel." It is more than that. It is taking off from the operator the armor of courage and confidence in his own ability to make his work a real blessing to his patient, and leaves him bare and unprotected against the temptation of doing hasty, botchy and inartistic work. The evil effects of this practice are not felt by the patient only, but indirectly by the operator, who becomes demoralized and ready to cut and hack with the reverence of a bull in a china closet, unmindful of the fact that nature, in her constructive processes, has produced in the teeth of man a marvelous example of the adaptation of means to ends—of means by which a given amount of tooth materal is distributed so as to seeure a combination of the greatest amount of strength with the greatest amount of beauty of form and outline. The marvelously beautiful curves and outlines that are shown by the teeth could have held the attention and fired the imagination of even Michael Angelo, who would have found in them examples of arches and abutments for his studies in architecture, and of models of grace and beauty of outline for his works of sculpture.

The cusps of the teeth suggest Gothic domes, and half of a cross section of a bicuspid is a perfect model of a Roman arch. If the enamel could be taken off the dentine as you would take a thimble of your finger, and could be viewed from the under side, it would present a series of arches and domes that might well serve as models in construction for the great temples of the world.

If the arches of a temple are made with bricks and stones that are laid so as to support the weight of the whole structure, it does not require a very lively imagination to see that the enamel prisms are also laid so that they may best support the weight of mastication. How long could it be expected that St. Peter's at Rome would stand, if on opposite sides a huge Arthur disk should cut away the side walls and parts of the dome? It would have been before this a crumbling ruin, as countless numbers of teeth are that have been so treated.

I venture the prediction that if Michael Angelo had been a dentist instead of one of the greatest artists and the greatest architect the world has ever known, he would never have sinned against himself by mutilating a tooth. Inevitably he would have been a contourist, for his great soul would never have been content except in the study and the reproduction of the perfect models that only nature —the source of all artistic inspiration—sets. You may say that it is absurd to speak of Michael Angelo in connection with the teeth; that he was a product of one of the great art periods of the world, and that this is a scientific age, and the teeth must be studied from the scientific and utilitarian stand-point. This is all true, aud yet in all candor I ask you where is there an instance in the whole range of art work where a close regard for the models that nature gives is followed by such utilitarian results as when the shapes of the teeth are restored. So that we must conclude that Dr. Webb did not without reason invoke so often the spirit of the great master.

To consider the subject further from this standpoint, it is safe to say that the public would not long tolerate an oculist who snips off a part of the eyelid in order to operate more easily on the eye; or a surgeon who, in amputating a finger, cuts off an arm; and yet it will sit down in a comfortable modern dental chair and let the teeth be cut and hacked, and will try to believe it is all right, because the operator has the reputation of being a splendid dentist.

The tooth might not be as valuable as the eyelid or as the arm but it was modeled by the same Great Designer, and the difference is in degree, and not in kind.

From the days when Dr. Dwinelle, in 1855, illustrated and described the method of restoring contours, and Drs. Atkinson and Varney, between 1860 and 1870, verified and extended that practice to the present day, many attempts have been made to follow it by men who have not always succeeded, because they have failed to see the full significance of a strict restoration of contours, and have not always prepared their cavities so as to get free margins, and have not always built their fillings out so that when finished the teeth shall have firm lateral support and the gum perfect protection.

To a certain extent, it is unfair to admit the testimony of such operators against contour-filling. The system of restorations is one that must be practiced thoroughly if it is practiced at all. Wherever contours are attempted, they should be made so as to touch at or near the grinding end of the tooth. Dr. Dwinelle puts the whole subject in a nutshell when he says: "The teeth should be made to impinge at their point of largest circumference." There can be no compromise here. If even a slight space be left between them at this point, food will crowd through and lodge against the gum, and cause all the annoyance of the worst kind of permanent separations. This is so important that I should go to the other extreme and say that it is unnecessary, and sometimes unwise, to fully restore teeth that, from the loss of a neighbor or a change in the occlusion, must eventually move slightly apart. I think it better to anticipate such inevitable and permanent change of position, and make at once a free parallel-sided space down to the gums; for such a space is less annoying than one wide at the gums and narrow at the grinding ends of the teeth.

You will see by this that I do not advocate contour for contour's sake. However much we may admire the form of the tooth, and however strong our desire to reproduce it, this must not be done if it is not to the advantage of the patient. The utilitarian side of the question must always be considered first. I wish to state this with great clearness, lest it may appear that my bias is for contour in order to keep alive my reverence for nature, and to satisfy my love of form and outline. It must be admitted that this parallel-sided space, so made that the teeth do not touch and the food does not become imparted against the gum, is one that, if it can be maintained permanently, gives a good result. It is the only separation between the bicuspids and molars that, from my standpoint of observation, can be justified. In effect, in a very modified way, it is like the extraction of a tooth. But the conditions do not often occur where it can be safely made. When free to move, the teeth migrate; and such a space as this must not be made unless the teeth will be held in place by the occlusion. Movement of the teeth is always in direction of least resistance; and if they are cut and allowed to move, not even an operator of long experience can always tell where they will be found after a few years have gone by. The cutting between sound teeth in anticipation of decay, so loudly lauded by some operators a few years ago, was a part of the Arthur system, and will die with it.

I have given some of the reasons why I am an advocate of the restoration of the shapes of the teeth. There are many more, and some of them are as important and convincing as any I have stated. In fact, there are so many reasons for that practice that unless I deny the logic of experience and reject the evidence of my senses, there can be no choice for me but to adopt it. I cannot believe that my bias has led me to see the subject out of its true proportions; for, whatever the practice may still be, I hear from all sides strong expressions of regret that Dr. Arthur's book was ever published. And, by the way, is it not a curious fact that a man who had been one of the first to discover the cohesive properties of gold, by the means of which the shapes of the teeth could be restored, should have advocated the permanent separation of them? But even his faith in that system was not so strong as his book, to a superficial reader, would imply; and I could quote from his letters to me to show that the book was written for the profession as he knew it, and not for it as it might and would be. He was too honest not to admit that, for those who could and would make restorations, that was the best practice.

Some years since, Dr. E. J. Dunning, one of the most accurate workers ever known to the profession, said to me that it was the regret of his life that he had ever cut the teeth so much. And only a few days ago I had the supreme satisfaction of being strengthened and confirmed in my faith in this practice by hearing Dr. Benj. Lord, whose conscientiousness and earnestness are known wherever the language is spoken, and whose enthusiasm for the art of saving teeth grows stronger and glows warmer with his advancing years, say that it was the regret of his professional life that he ever fell into the practice of cutting away and shaping proximate surfaces in order to secure permanent separations after filling, and also that he advocated as well as practised filing and shaping the teeth to prevent decay.

I have had deep feeling on this subject for .a great many years, and you must pardon me if I give utterance to the satisfaction I feel when I hear such words as these, and you must let me further express the pleasure I feel in knowing that Drs. Dwinelle and Atkinson, pioneers in this work, are here with us, spared to see the day when the irresistible tide of ripened professional judgment sets in their direction.

If I have spoken severely of the injuries done to the teeth by the system of permanent separations, I must not overlook the fact that it is a system that has some merit, otherwise it never could have been so long and so widely practiced. There can be no question that innumerable numbers of teeth have been saved by it; but those were the ones that ran the gauntlet and escaped the danger; and, after all, it is this risk and danger that I want to see our profession freed from.

We must also be careful not to allow a suggestion of harshness in speaking of the early operators who practiced the system. They did the best they could. They did not have the conveniences and appliances of the present day; and, moreover, sufficient time had not elapsed for them to see the evil effects of that practice. Dr. Arthur, who did more than any other man to establish the system, must be spoken of only in terms of respect, for he did in his day and in his way only what he thought was best for the profession. Nothing would be more ungracious than to speak in any but the most charitable terms of these operators, who did not and could not do in early professional life what can be easily accomplished at the present day. If we attempt to measure by too high a standard, where shall we find one of us who will not be too short. Let us overlook the past and be thankful for the bright future before us, remembering always that to be charitable and helpful to each other is to do the most to advance the cause of our beloved profession.

There is still another side to the subject that must not be allowed to pass without consideration. Partly by its own impulse the pendulum swings to the other side of its arc, and some of the best operators, repelled by the evils of permanent separations, have gone to the other extreme, and in their eagerness to restore the teeth have cut them away far more than necessary, and have given themselves and their patients needless trouble in restoring them. In my judgment this was an error constantly made by both Drs. Varney and Webb. If this can be justly said of those accurate workers of gold, with how much more force does it apply to those who follow the same plan, but are not such accurate operators? I am fully convinced that it is an error which I have many times committed, and it is one that any one may easily fall into if they have not come to have what I will venture to call reverence for the natural teeth. It is one that has been encouraged by the use of the dental engine and by the use of cohesive gold. A graduate of one of our colleges came to me with hardly a dozen excavators, and those of the most useless kind, in his outfit. He prepared his cavities mainly with the dental engine. It is not surprising that a man like Maynard refuses to believe that modern dentistry has made the progress claimed for it. A disheartening picture could be drawn if one felt inclined to contemplate the injury that has been done by confining operations on the teeth within such narrow lines. To ignore the advantages of soft foil and to be unskilled in its use is a misfortune to the patient, to the dentist and to the profession. The man is dwarfed who is not as ready to apply one system as the other.

It would be better for our patients and better for dentistry if we could keep more in mind the idea that the human teeth are marvelous structures and worthy of the most patient and pains-taking efforts of the most accomplished men in the world.

And whatever method is employed they never will be saved without this painstaking care. While the physical properties of gold remain what they are, it is useless to hope for any easy method of filling with it large cavities on the proximate surfaces of the bicuspids and molars. It always has been and always will be absolutely impossible to get good results without hard work, so that in selecting the method we may as well dismiss at once the idea of finding an easy one. The search for such a one has resulted in an immense amount of bad work. The search for a rapid one is equally misleading. Under the pressure of a large practice it has done more to keep my own work below the standard I could wish to work to, than all other causes combined. If plastics are used the same patient care is necessary, if we are to get good results. In the very nature of things there can be no easy way to permanent success of any kind.—*International Dental Journal.*

